# Asymmetry of the sulcal pattern of the anterior cingulate cortex modulates delay discounting

**DOI:** 10.1007/s00429-026-03114-8

**Published:** 2026-04-27

**Authors:** Federica Santacroce, Federica Procida, Antonello Baldassarre, Arnaud Cachia, Carlo Sestieri, Giorgia Committeri

**Affiliations:** 1 Department of Psychology (DiPSI), University “G. D’Annunzio” of Chieti -Pescara, 66100 Chieti , Pescara Italy; 2 Department of Neuroscience, Imaging and Clinical Sciences, University G. d’Annunzio of Chieti-Pescara, Via dei Vestini 31, 66100 Chieti , Pescara Italy; 3 ITAB Institute for Advanced Biomedical Technologies, University G. d’Annunzio of Chieti-Pescara, Via dei Vestini 31, 66100 Chieti, Pescara Italy; 4 Laboratoire de Psychologie du développement et de l’Education de l’Enfant (LaPsyDÉ), Université Paris Cité, CNRS UMR 8240, 75005 Paris, France; 5 Université Paris Cité, Institut de Psychiatrie et Neurosciences de Paris (IPNP), INSERM, UMR S1266, 75014 Paris, France; 6 CARES Centre for Disability, Rehabilitation and Sports Medicine, Viale Abruzzo, 322, 66100 Chieti, Pescara Italy

**Keywords:** Delay discounting, Morphology, Anterior cingulate cortex, Sulci, Asymmetry, Human connectome project, Brain disorders

## Abstract

Delay discounting (DD), the preference for smaller immediate over larger delayed rewards, is associated with impulsivity and cognitive control, representing a transdiagnostic process across different neuropsychiatric disorders. While previous neuroimaging studies have mainly focused on the functional neural substrates of DD, their anatomical correlates have received less attention. Based on accumulating evidence about the role of cortical sulci morphology as an indirect marker of fetal neurodevelopment, we examined the relationship between DD and the sulcal pattern of the anterior cingulate cortex (ACC), a cortical area classically associated with DD. In a large sample of young healthy adults (*N* = 390, 173 males), extracted from the Human Connectome Project (HCP) dataset, we defined the ACC sulcal pattern of each hemisphere as ‘single’ or ‘double parallel’ based on the absence or presence of the paracingulate sulcus (PCS), and then classified each individual brain as symmetric or asymmetric across the two hemispheres. DD rates were obtained from a Principal Component Analysis (PCA) of the results of a behavioral battery administered to the same participants. To ensure the specificity of the findings, statistical models were controlled for age, gender, and other cognitive factors, also derived from the PCA, as well as compared with the pattern of a different sulcus. Individuals with an asymmetric ACC sulcal pattern (i.e., inter-hemispheric differences in pattern type) exhibited lower discount rates, indicating a greater preference for delayed rewards, compared to those with a symmetric pattern. In the PCA-based analysis, the association significantly involved the factor capturing DD. Additional analysis on individual tasks supports this preferential relationship but further revealed an association between ACC morphology and processing speed. No comparable association was observed for the morphology of the Intra-Parietal Sulcus (IPS), used as a control region. Since sulcal patterns are determined during fetal life, our findings support a neurodevelopmental basis for individual differences in intertemporal choice and provide insights into the structural predisposition to impulsivity-related traits and their associated clinical pathologies.

**Supplementary Information:**

The online version contains supplementary material available at 10.1007/s00429-026-03114-8.

## Introduction

Delay discounting (DD) refers to the tendency towards smaller immediate over larger delayed rewards. Indices of discounting, such as the discounting rate (*k value*), are typically derived from tasks in which participants choose between small or large rewards that can be delivered sooner or later, respectively (Impulsivity 2010). Given its implication in several disorders - including substance abuse (Amlung et al. 2017), gambling (MacKillop et al. 2014), and obesity (McClelland et al. 2016)- DD has been proposed as a transdiagnostic factor (Amlung et al. 2019, Bickel et al. 2012, Bickel and Mueller 2009, Levin et al. 2018). Reward-related decision-making may therefore represent a therapeutic target for the treatment of these disorders (Cona et al. 2019). In this framework, DD has been extensively studied as a potential marker of cognitive dysfunction across various clinical conditions, providing valuable insights into the neural and psychological mechanisms underlying maladaptive choice behavior (Chesson et al. 2006, Kirby et al. 2005, Meier and Sprenger 2012). Furthermore, research on DD has been instrumental in elucidating the complex cognitive mechanisms underlying human decision-making, which requires a dynamic balance between valuation, executive control, and future-oriented thinking.

Neuroimaging studies have attempted to uncover the neural correlates of DD. The results from functional neuroimaging studies converge on the role of three brain networks (Lempert et al. 2019): (1) a ventral cortico-striatal network for reward valuation, including the ventral striatum and the ventromedial prefrontal/orbitofrontal cortex (Peters and Büchel 2010); (2) a medial network for prospective evaluation of future outcomes, containing the medial temporal cortex, the posterior cingulate/precuneus, and regions of the dorsomedial prefrontal cortices (Monterosso et al. 2007, McClure et al. 2004, Peters and Büchel 2011); and (3) a lateral prefrontal-cingulate network for executive control, comprising the dorsolateral prefrontal cortex (dlPFC) and the anterior cingulate cortex (ACC). This last network mediates the engagement of self-control mechanisms required to suppress impulsive tendencies in favor of long-term benefits. Specifically, the interaction between the two main nodes of the lateral prefrontal-cingulate network (dlPFC and ACC) plays a key role in conflict monitoring and effort-based decision-making, especially when the trade-off between immediate and delayed rewards is less clear (Lempert et al. 2019, Koban et al. 2023). A meta-analysis of 78 fMRI studies further corroborated these findings, highlighting the consistent activation of the three networks across different intertemporal choice paradigms (Souther et al. 2022).

More recently, structural neuroimaging studies have started to examine the structural underpinning of DD through the analysis of cortical thickness (Sadeh et al. 2023), grey matter volume (Wang et al. 2017), and white matter integrity (Han et al. 2018, Olson et al. 2009). Variations in the bilateral ACC grey matter volume (Li et al. 2019) and right inferior frontal sulcus depth (McIntyre-Wood et al. 2022) have been associated with inter-individual differences in discount rates. However, since these quantitative measures are plastic and change over time as a result of physiological aging, learning, or the onset of certain diseases (Armstrong et al. 1995, Casey et al. 2000, Tudor et al. 2021), it is difficult to determine whether anatomical changes precede the development of DD or are a neuroplastic consequence of this phenomenon.

To overcome these limitations, here we analyzed a qualitative feature of the cortical mantle, namely the cortical sulcal pattern, which is established during fetal development and remains stable throughout the lifespan (Cachia et al. 2021). Indeed, unlike quantitative cortical features, sulcal pattern is determined during fetal life (between 10 and 15 weeks for the ACC) and is already evident at birth, in a configuration that mirrors that of adulthood (Chi et al. 1977, Mangin et al. 2010, Zilles et al. 2013). Moreover, the morphological pattern does not change over time, as established by longitudinal studies (Cachia et al. 2016, Schwizer Ashkenazi et al. 2024, Tissier et al. 2018). In light of these peculiarities, the influence of sulcal pattern on cognition has drawn increasing attention from researchers in recent years, supporting the idea that particular morphological patterns may be advantageous for cognitive development (Cachia et al. 2021, Tissier et al. 2018, Santacroce et al. 2024), thereby representing a positive neural fingerprint. Several studies have investigated the anterior cingulate cortex (ACC) and its two morphological variants, defined by the presence (“double parallel pattern”) or absence (“single pattern”) of the paracingulate sulcus (PCS), which runs dorsal and parallel to the (always present) cingulate sulcus. In particular, the asymmetry of the ACC pattern (e.g., a double parallel pattern in one hemisphere and a single pattern in the other) has been repeatedly associated with better performance on cognitive control tasks, both in adults (Tissier et al. 2018, Fornito 2004, Huster et al. 2009) and children (Borst et al. 2014, Cachia et al. 2014).

Given the role of the ACC in executive/cognitive control (Alvarez and Emory 2006, Petersen and Posner 2012) and its involvement in DD tasks (Schüller et al. 2019, Seamans et al. 2024), we hypothesize that the stable morphological features of the ACC might influence individual differences in intertemporal choice behaviors. Building on this premise, our study examined the relationship between ACC sulcal morphology and delay discounting in a large sample of healthy young adults (*N* = 390) provided by the Human Connectome Project (HCP). We hypothesized that individuals with an asymmetric ACC sulcal pattern would exhibit lower discount rates, i.e., greater preference for delayed rewards, compared to those with a symmetric pattern. The specificity of this relationship was assessed by controlling for the effect of a different sulcus (i.e., the intraparietal sulcus, IPS) along with other cognitive measures (i.e., memory and language, other indices of executive functions). Moreover, although we aimed to study the sulcal morphology of the ACC, we further investigated the morphological variability of the cingulate sulcus (CS) itself, which covers a wider region, extending further posteriorly toward the parietal lobe.

By focusing on stable, in utero-determined, morphological features, the present research represents a first step to elucidate whether steady structural configurations of the ACC reflect a neural predisposition able to influence the tendency to devalue future rewards across the lifespan. Understanding this relationship may have important implications for interventions targeting cognitive control mechanisms in neuropsychiatric disorders characterized by impulsive decision-making.

## Methods and materials

### Participants

Participants were extracted from the 1200 Subjects Release (S1200) of the Human Connectome Project (HCP) dataset, with the following inclusion criteria: age range: 26–30 years old; MRI field: 3 T, DTI data (not analyzed for this study). Participants with quality control issues.

(https://humanconnectome.org/storage/app/media/documentation/s1200/HCP_ S1200_Release_Reference_Manual.pdf), major neurological diseases and psychiatric or medical disorders were excluded. A total of 390 participants (173 males, 44%) were included in the study. Participants gave informed consent, and all recruitment and acquisition methods were approved by the Washington University Institutional Review Board (IRB), following relevant guidelines and regulations.

## Cognitive measures

The behavioral indices used in this study were derived from a Principal Component Analysis (PCA) performed by Santacroce and colleagues (2024) on all cognitive measures available in the HCP database (18 behavioural measures/metrics derived from 12 different cognitive tasks within 8 general cognitive domains). In particular, as a measure of DD, we used the third factor of the PCA, which explained 8.9% of the total behavioural variance and captured two self-regulation/impulsivity tasks typically used to assess delay discounting (https://humanconnectome.org/storage/app/media/documentation/s1200/HCP_S1200_Release_Reference_Manual.pdf, Chapter 5, pages 193–195). In the two self-regulation/impulsivity tasks, DD was measured using the area under the curve (AUC) approach, which quantifies an individual’s tendency to discount delayed rewards (Green and Myerson 2004, Myerson et al. 2001). In particular, the AUC reflects the subjective decline in the value of delayed rewards over time, with smaller areas indicating greater discounting and larger areas indicating weaker discounting. The task followed an adjustment amount paradigm (Estle et al. 2006) in which fixed reward delays were paired with dynamically adjusted reward amounts until an indifference point was reached, representing the delay at which a participant equated a smaller immediate reward with a larger delayed reward. Participants chose between a smaller immediate reward and a larger delayed reward across six delays (1 month, 6 months, 1 year, 3 years, 5 years, 10 years) and two reward amounts ($200; $40,000). Each condition consisted of five trials, with the final choice determining the indifference point. On the first trial, the immediate reward was set at 50% of the delayed amount and adjusted up or down based on the participant’s choice. Subsequent adjustments followed a fixed proportion of the previous change, rapidly converging on the indifference point for each delay-reward pair. These points were then used to calculate the AUC for each reward level.

As behavioural control measures to assess the specificity of the relationship between ACC morphology and DD, we used the first (‘Memory and Language’) and second (Executive Functions’) factors of the PCA, which explained 20.6% and 12.1% of the behavioural variance, respectively.

## MRI acquisition and pre-processing

The 3 T anatomical MRI included in the HPC dataset corresponded to T1-weighted images acquired on Siemens 3 T “Connectome Skyra” scanner using a 3D Magnetization Prepared Rapid Acquisition Gradient Echo (MPRAGE) sequence (TR = 2400 ms; TE = 2.14 ms; TI = 1000 ms; flip angle = 8°; FOV = 224 × 224 mm; voxel size = 0.7 mm isotropic). The MRIs were preprocessed using the standard FreeSurfer pipeline used in HCP (Glasser et al. 2016). T1 MRIs were volumetrically registered to the MNI152 space using nonlinear FNIRT followed by surface registration to Conte69 ‘164k_fs_LR’ mesh (Essen et al. 2012), with FreeSurfer fsaverage as an intermediate.

Based on each participant’s MRI scan, a 3D model of the cortical surface was reconstructed using FreeSurfer 5.2. This 3D model included the segmentation of the white matter, the tessellation of the grey/white matter boundary, the inflation of the folded, tessellated surface, and the correction of topological defects.

## Characterization of the sulcal pattern

Following the same approach developed in our previous work (Santacroce et al. 2024), the sulcal maps were superimposed on the corresponding highly inflated and pial surfaces for each participant to visualize the morphology of the ACC, using the Connectome Workbench visualization software. The sulcal pattern of the dorsal ACC was characterized according to Ono’s classification (Ono et al. 1990) by visually inspecting each individual’s 3D mesh-based reconstruction. All MRI data were anonymized, and the ACC sulcal pattern was classified by two raters (FS and FP). Sulcal pattern labeling was performed blind to potential confounds such as participants’ age, label in the contralateral hemisphere, and previously labeled IPS sulcal pattern. After that, all discrepant or doubtful cases (*N*  were re-examined by a third expert (GC). 

**Fig. 1:**
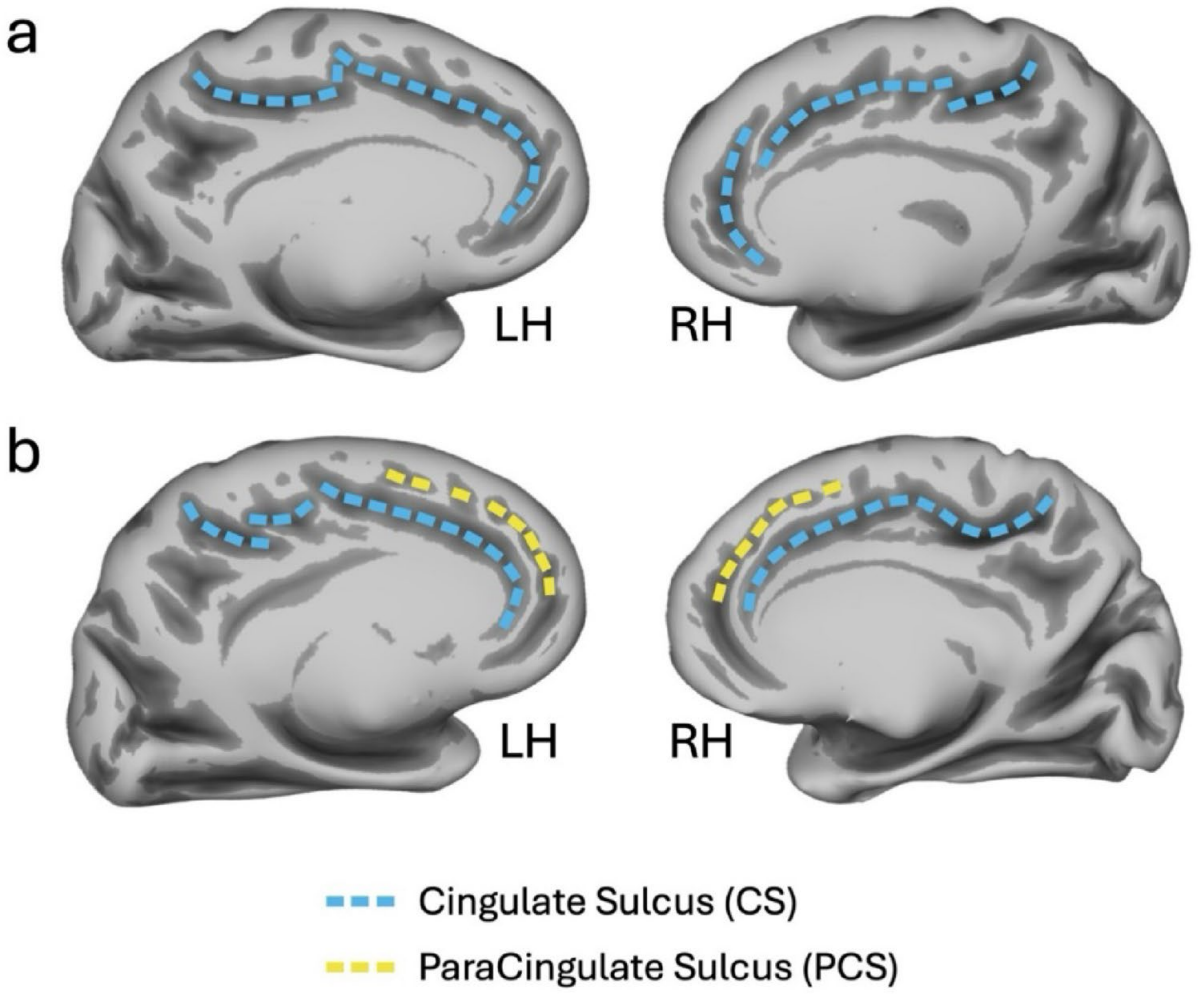
Sulcal pattern of the anterior cingulate cortex (ACC). **a** Exemplars of single patterns in which only cingulate sulcus is present (sulci shown with cyan dashed lines). **b **Exemplars of double-parallel patterns in which both cingulate (sulci shown with cyan dashed lines) and paracingulate (sulci shown with yellow dashed lines) sulci are present. LH left hemisphere, RH right hemisphere

The ACC sulcal pattern was classified into two anatomical variants according to Ono’s classification (Ono et al. 1990)(“single type” or “double parallel type”), based on the absence/presence of the paracingulate sulcus (PCS), respectively (see Fig. 1 for representative examples). The PCS, when present, was located dorsally to the cingulate sulcus and ran parallel to it (Paus et al. 1996). To disambiguate the confluence of the PCS with the superior rostral sulcus (SRS), we defined the anterior border of the PCS as the point where the sulcus extended posteriorly beyond an imaginary vertical line perpendicular to the axis connecting the anterior and posterior commissures (AC-PC) (Yucel et al. 2001). The PCS was only considered to be present if it extended at least 20 mm horizontally beyond this imaginary vertical line. The sulcal pattern was considered “symmetric” if it was the same in both hemispheres and “asymmetric” if it differed between the hemispheres. In addition, we characterized the variability of the cingulate sulcus (CS) with respect to sulcal interruptions. Details regarding this further morphological classification are reported in the Supplementary Materials.

The procedure for the characterization of the IPS, used as a control sulcus in the present study, has been described in detail in our previously published study (Santacroce et al. 2024). Briefly, IPS morphology can have two different sulcal patterns: the “interrupted pattern,” when the course of IPS showed one or more interruptions by a cortical gyrus, and the “continuous pattern,” when no interruptions were detected. Since our previous study already demonstrated that neither the right nor the left hemisphere IPS pattern influenced DD, here we further control for the IPS symmetric/asymmetric pattern to ensure that the effect of cortical morphology on delay discounting is not generalized but specifically related to the ACC. Similar to the ACC, the symmetry/asymmetry IPS sulcal pattern was defined as “symmetric” if the sulcal pattern was identical in both hemispheres and “asymmetric” if it differed between the hemispheres.

### Statistical analysis

Based on the classification of the ACC pattern in each participant, an exact binomial test with exact Clopper-Pearson 95% CI was performed to examine the difference in distribution between the “single” and “double parallel” patterns in the left and right hemispheres and between the symmetry and asymmetry distributions. To compare the distribution of the ACC pattern and its left-right asymmetry in our sample versus that obtained in previous studies with healthy adults (Tissier et al. 2018, Yucel et al. 2001) and children (Tissier et al. 2018, Borst et al. 2014, Cachia et al. 2014), we performed a chi-square (χ²) test with post-hoc pairwise comparisons, adjusted by Bonferroni correction, controlling for the sample size.

We then investigated the association between ACC individual morphological variability and DD rate using a Generalized Linear Model (GLM) including the ACC pattern symmetry/asymmetry and sex as categorical factors and the third factor of the PCA (delay discounting) as dependent variable. Sex was included in the analysis because of its effect on cortical morphology (Duchesnay et al. 2007, Fish et al. 2016). Detailed analyses and results on the potential effect of the CS interruptions and their symmetry/asymmetry are reported in the Supplementary Materials.

To test the specificity of the ACC/DD relationship, we performed a similar GLM, adding the symmetrical/asymmetrical pattern of the IPS as further morphological variable and the first two factors of the PCA (memory and language, executive functions) as further behavioral variables. Specifically, the symmetrical/asymmetrical pattern of the ACC and the symmetrical/asymmetrical pattern of the IPS were included in the control GLM as fixed factors, and the first (memory and language), second (executive functions), and third (delay discounting) factors of the PCA as the dependent variables.

For completeness of analysis, we performed an additional single GLM entering all individual PCA metrics/tasks as dependent variables in relation to ACC morphology and sex as independent variables. The results are presented in Table S2 of the Supplementary Materials.

## Results

### ACC sulcal pattern distribution and comparison with previous studies

The inter-rater reliability yielded an agreement of 92.82% regarding the presence of the single vs. double-parallel ACC pattern for both the left and right hemispheres. After that, all discrepant or doubtful cases (*N* = 28) were re-examined by a third expert (GC). This process ensured a final agreed-upon label for every doubtful case, such that no commonly rated case remained unclear and no participant was excluded.

The ‘single’ type was found in 81.28% of the right hemispheres and 53.85% of the left. Our sample was nearly evenly divided between symmetrical and asymmetrical patterns, with 51.54% being symmetrical and 48.46% being asymmetrical.

**Fig. 2:**
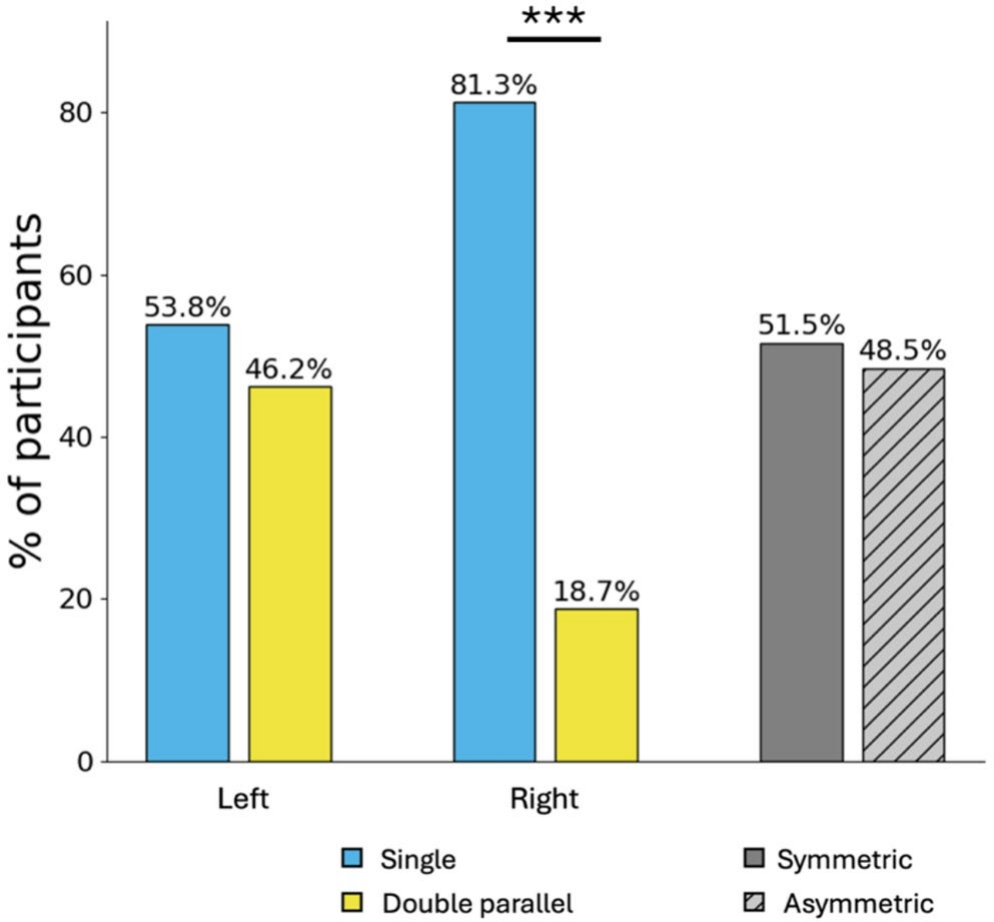
Sample distribution of the ACC sulcal pattern. Percentage of participants with single (in cyan) and double parallel (in yellow) patterns in both left and right hemispheres, and percentage of the same participants divided into symmetrical (dark grey) and asymmetrical (light grey with oblique lines) patterns. ***p < 0.001; sample size *n* = 390

A binomial test showed a significant difference in the number of “single” and “double parallel” ACC patterns in the right hemisphere (*p* < 0.001, 95% CI of 77.4%−85.1%), the “single” being more represented than the “double parallel” type. In contrast, there was no significant difference in the proportion of “single” and “double parallel” ACC patterns in the left hemisphere (*p* = 0.14, 95% CI of 49.0%−58.7%) and no significant difference between symmetric and asymmetric ACC (*p* = 0.57, 95% CI of 46.7%−56.4%). The ACC pattern distributions are shown in Fig. 2.

**Fig. 3:**
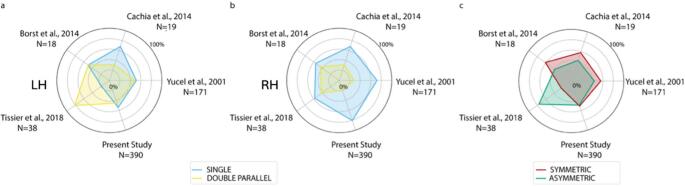
Comparison with previous studies. Comparison between the present and previous studies of ACC sulcal pattern sample distribution. The cyan lines represent the “single” pattern and the yellow lines the “double-parallel” pattern, in the **a** left (LH) and **b** right (RH) hemispheres. The red line represents the “symmetric” pattern and the green line the “asymmetric” pattern (**c**)

The chi-square (χ²) analysis, controlling for the sample size, revealed a significant difference among the five studies regarding the frequency distribution of the left (χ²= 53.6998, df = 4, *p* < 0.001) and right (χ²= 20.0947, df = 4, *p* < 0.001) ACC pattern as well as of the ACC symmetry/asymmetry (χ²= 36.7340, df = 4, *p* < 0.001) (see Fig. 3; Table 1 for details). Post-hoc pairwise comparisons showed that in the study by Tissier and colleagues (Tissier et al. 2018), the frequency of the double pattern of the left ACC was higher than that observed in all other studies (all *p* < 0.001). For the right hemisphere, Tissier’s and Borst’s samples showed a more balanced distribution of the two patterns than in the present study (both *p* < 0.001). Regarding the distribution of symmetry/asymmetry, Tissier’s cohort showed a higher prevalence of the asymmetric pattern than all other studies (all *p* < 0.001). Details of post-hoc pairwise comparisons are reported in the Supplementary Table S1.

**Table 1:** Frequency distribution of left and right ACC pattern and ACC symmetry/asymmetry pattern in the present and previous studies

	Study	Pattern	N (%)	chi-square
Left ACC	Yücel et al., 2001 (*N* = 171)	Single	88 (51%)	χ²=53.7585 df = 4 *p* < 0.001
		Double-parallel	83 (49%)	
	Cachia et al., 2014 (*N* = 19)	Single	13 (68%)	
		Double-parallel	6 (32%)	
	Borst et al., 2014 (*N* = 18)	Single	9 (50%)	
		Double-parallel	9 (50%)	
	Tissier et al., 2018 (*N* = 38)	Single	7 (18%)	
		Double-parallel	31 (82%)	
	Present study (*N* = 390)	Single	210 (54%)	
		Double-parallel	180 (46%)	
Right ACC	Yücel et al., 2001 (*N* = 171)	Single	123 (72%)	χ²=20.0947 df = 4 *p* < 0.001
		Double-parallel	48 (28%)	
	Cachia et al., 2014 (*N* = 19)	Single	13 (68%)	
		Double-parallel	6 (32%)	
	Borst et al., 2014 (*N* = 18)	Single	10 (56%)	
		Double-parallel	8 (44%)	
	Tissier et al., 2018 (*N* = 38)	Single	22 (58%)	
		Double-parallel	16 (42%)	
	Present study (*N* = 390)	Single	317 (81%)	
		Double-parallel	73 (19%)	
Symmetry/asymmetry ACC	Yücel et al., 2001 (*N* = 171)	Symmetric	96 (56%)	χ²=36.7060 df = 4 *p* < 0.001
		Asymmetric	75 (44%)	
	Cachia et al., 2014 (*N* = 19)	Symmetric	11 (58%)	
		Asymmetric	8 (42%)	
	Borst et al., 2014 (*N* = 18)	Symmetric	11 (61%)	
		Asymmetric	7 (39%)	
	Tissier et al., 2018 (*N* = 38)	Symmetric	9 (24%)	
		Asymmetric	29 (76%)	
	Present study (*N* = 390)	Symmetric	201 (51%)	
		Asymmetric	189 (49%)	

## Relationship between ACC symmetry/asymmetry and delay discounting

The relationship between the ACC symmetry/asymmetry and the DD rating was assessed using a Generalized Linear Model (GLM) including the ACC symmetry/asymmetry (“symmetric” vs. “asymmetric”) and sex (“males” vs. “females”) as categorical factors and the third factor of the PCA (reflecting the delay discounting measure) as the dependent variable.

**Fig. 4:**
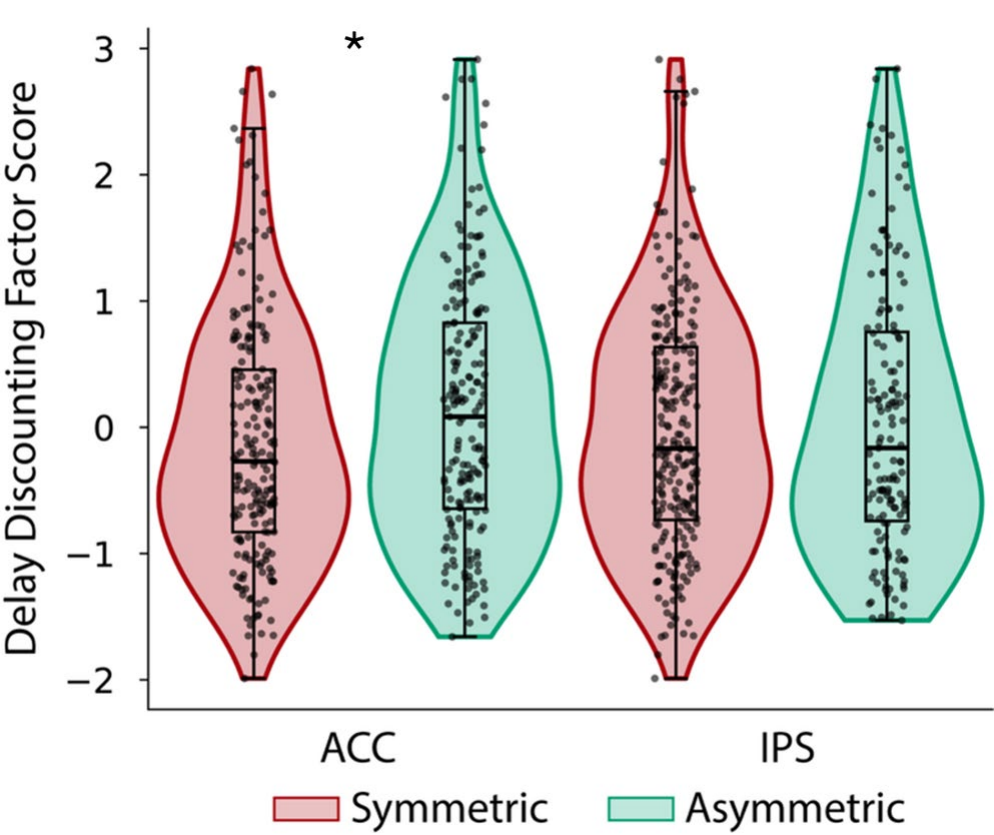
ACC symmetry/asymmetry pattern and Delay Discounting. Violin and box plot with individual data points represent the scores of the Delay Discounting Factor in participants with symmetric (red box) and asymmetric (green box) ACC (left boxes) and IPS (control sulcus, right boxes). Boxes indicate the interquartile Range (IQR, data points included between the first quartile, 25th percentile, and the third quartile, 75th percentile); horizontal black lines indicate the median (50th percentile). The whiskers go from the minimum to the lower quartile (the start of the box) and then from the upper quartile (the end of the box) to the maximum. *p < 0.05; sample size *n* = 390

This analysis revealed a main effect of the ACC sulcal pattern asymmetry (β = 0.2117, df = 1, *p* = 0.036), whereas no effect was found for sex (β= −0.01510, df = 1, *p* = 0.137). In particular, an asymmetric pattern of ACC was associated with a higher AUC, indicating a less steep discounting of delayed rewards (see Fig. 4).

To test the specificity of the ACC effect on the DD measure, we (1) added a categorical factor related to the IPS asymmetry (“symmetric” vs. “asymmetric”) in the previous GLM and (2) also analyzed the first (memory and language) and the second (executive functions) factor of the PCA as a dependent variable. The analysis confirmed the main effect of ACC symmetry/asymmetry on DD (β = 0.2059, df = 1; *p* = 0.042), but no significant results were found for the two additional PCA factors (both *p* > 0.05). Again, an asymmetric pattern of the ACC was associated with a higher AUC. Conversely, the symmetry/asymmetry of the IPS pattern did not affect DD (see Fig. 4), demonstrating a morphological dissociation (*p* = 0.519). Finally, there was no significant effect of the IPS symmetry/asymmetry on the first (β= −0.0254, df = 1, *p* = 0.809) and second (β= −0.1301, df = 1, *p* = 0.209) factors of the PCA.

## Discussion

The present study provides the first evidence that a stable, in utero-determined morphological feature of the anterior cingulate cortex (ACC) is associated with interindividual differences in delay discounting (DD) behavior. Our results indicate that individuals with an asymmetric ACC sulcal pattern are less prone to discounting future rewards compared to those with a symmetric pattern, suggesting a greater ability to value delayed outcomes. This finding is possibly due to enhanced cognitive control mechanisms, consistent with previous evidence linking ACC asymmetry to better cognitive control. Notably, this effect was not observed in relation to other cognitive domains, including memory and language, or morphological features (the symmetry of the interruption pattern of either the CS or the IPS).

Our findings are consistent with previous studies that highlight the ACC as a critical hub in the brain network of decision making, mediating conflict monitoring, effort allocation, and reward evaluation (Botvinick et al. 2004, Shenhav et al. 2013), all aspects that are central to discounting behavior. Such cognitive functions, which require the involvement of many high-level processes, need to be viewed from a network perspective. The main effect of ACC asymmetry on DD observed in the present study underscores the importance of inter-hemispheric communication, and particularly the critical role of structural brain asymmetries in shaping intertemporal decision-making. Specifically, our results show that individuals with an asymmetric ACC sulcal pattern exhibit a higher area under the curve (AUC) in DD tasks, indicating a more patient evaluation of delayed rewards compared to individuals with a symmetric ACC pattern. This suggests that the structural hemispheric asymmetry within the ACC may shape the development of cognitive control mechanisms, supporting the ability to prioritize long-term benefits over immediate gratification. Conversely, the presence of hemispheric symmetry may impose constraints on the development of efficient cognitive control mechanisms, potentially limiting the ability to delay gratification.

The presence of structural asymmetry in the ACC is likely to reflect intra-hemispheric specialization, reducing duplication and improving neural efficiency (Corballis and Left Brain 2014), as information transfer is more efficient between nearby areas within the same hemisphere than between distant regions across both hemispheres (Deary et al. 2010, Toga and Thompson 2003). The asymmetry of the ACC may foster the effective integration between reward valuation, e.g., through intra-hemispheric connections with the limbic system and vmPFC (Behrens et al. 2009, Rushworth et al. 2012), and behavioral regulation, e.g., through intra-hemispheric connections with the dlPFC (Kanske et al. 2011, Miller and Cohen 2001). This may explain why individuals with an asymmetric ACC sulcal pattern show a greater ability to resist impulsive choices efficiently and to evaluate delayed rewards more promptly, a cognitive advantage that may be linked to associations between cortical folding morphology and the brain’s underlying structural connectivity (Essen 1997, Hilgetag and Barbas 2006, Essen 2020). On the other hand, the lack of a significant relationship between ACC morphology and other cognitive measures not typically associated with the ACC (i.e., memory and language) reinforces the evidence that the effects of ACC sulci asymmetry is preferential for DD.

Indeed, when ‘Executive Functions’ were examined together with the ‘Memory and Language’ factor, the ACC–DD association remained the most prominent effect in our dataset. The lack of a corresponding association with the factor named “executive functions” (Factor 2) may appear unexpected. However, this result might be explained by the intrinsic heterogeneity of the executive domain, which aggregates partially dissociable processes (e.g., inhibition, cognitive flexibility, planning). While we acknowledge that the behavioral battery of the HCP did not systematically cover the executive domain, the pattern of ACC may be only relevant for some, but not all, executive aspects and tasks. Consistent with this interpretation, previous studies have linked ACC sulcal patterns to Stroop Task and the Trail Making Test (TMT) in patients suffering from schizophrenia (e.g. Tissier et al. 2018, Gay et al. 2017), but not to other executive measures like working memory (Borst et al. 2014). Moreover, when considering individual tasks outside the PCA framework (see Table S2), we also observed an association between ACC symmetry/asymmetry and processing speed, a core ability strictly linked to executive functioning (Vanhala et al. 2023) and indeed included in the second “executive function” PCA factor (Santacroce et al. 2024). This indicates that the relationship between ACC morphology and cognition is not strictly limited to delay discounting at the single-task level. However, in the PCA-based analysis, the effect emerged only for the latent factor capturing delay discounting, suggesting that ACC symmetry/asymmetry may be more closely related to the shared variance underlying decision-making processes rather than to that shared by different executive processes.

Notably, we also found that the IPS pattern, a cortical region not directly involved in DD, did not affect delay discounting performance. Interestingly, this finding supports a double dissociation framework, whereby the morphology of regions not involved in DD (such as IPS) fails to predict DD performance, but also the morphology of DD-related regions (e.g., ACC) does not predict unrelated cognitive functions (e.g., memory and language). Notably, few studies examining the relationship between morphology and cognition have controlled for either other cortical regions or cognitive domains: some studies have focused on the morphological control (e.g., Occipito-Temporal sulcus) (Tissier et al. 2018), others on the cognitive control (e.g., verbal working memory) (Borst et al. 2014, Cachia et al. 2014). This is the first study to simultaneously test both morphological and behavioral specificity in a single, large sample. To enhance the robustness of this emerging field, it will be essential for future research to investigate the bidirectional specificity of brain-behavior relationships, ensuring that particular structural configurations are more or less selectively mapped to distinct cognitive profiles.

The confirmation of such a relationship would also allow for extending the present findings to personalized clinical applications aimed at improving intertemporal decision-making. For example, non-invasive neuromodulation techniques that are widely used in the treatment of dysfunctional choice behavior (e.g., addiction and gambling disorders), such as transcranial magnetic stimulation (TMS), could be adapted differently according to ACC morphology, potentially improving their ability to delay actuation. Indeed, several studies have found positive effects on delay discounting measures in patients suffering from addiction disorders through stimulation of the dlPFC (Zack et al. 2016, Pettorruso et al. 2021). Due to its medial location, the ACC is not an obvious target for stimulation. However, through its functional connection to the dlPFC, the morphological pattern of the ACC could likely influence the outcome of such treatments. It would also be interesting to exploit alternative neuromodulation techniques, such as the transcranial ultrasound stimulation (TUS) (Attali et al. 2025), which is more effective in targeting deep brain regions compared to TMS.

From an anatomical point of view, we observed that the distribution of the ACC single and double parallel patterns is balanced in the left hemisphere, consistent with previous studies (see Borst et al., 2014, Cachia et al., 2014, Yucel et al., 2001) (Borst et al. 2014, Cachia et al. 2014, Yucel et al. 2001), except for Tissier et al., 2018, which shows discordance with all other studies. In the right hemisphere, we observed that the single pattern is significantly more frequent than the double parallel pattern, contrary to what reported in previous studies (see Tissier et al., 2018, Borst et al., 2014) (Tissier et al. 2018, Borst et al. 2014). Finally, the interstudy comparison revealed discrepancies in the distribution of the asymmetric ACC pattern between the study by Tissier et al., 2018 and all other studies (see Borst et al., 2014, Cachia et al., 2014, Yucel et al., 2001) (Borst et al. 2014, Cachia et al. 2014, Yucel et al. 2001), as well as between the study by Tissier et al., 2018, Borst et al. 2014 and the present study. As shown in Fig. 3, studies with smaller sample sizes tend to deviate more from the others, to varying degrees, as previously discussed.

We suggest that the small sample size may introduce more random variability into the results, leading to inter-studies discordance, particularly when comparing studies with limited cohorts to larger samples such as those used in our study (*N* = 390) and in the study by Yucel et al., 2001 (*N* = 171). It is indeed well established that detecting small genetic effects associated with complex traits, such as cortical morphology, requires large sample sizes (Meer and Kaufmann 2022), particularly when examining these traits in relation to behavior (Marek et al. 2022). In addition to sample size, discrepancies across studies may also partly reflect methodological heterogeneity in sulcal labeling and reliability assessment (e.g., number of raters, reliability metrics, and adjudication strategies), which can introduce measurement noise and affect the reproducibility of structure-behaviour associations. These considerations underscore the importance of conducting studies with sufficiently large sample sizes to reliably capture stable interindividual variability in cortical morphology and to ensure the reproducibility of findings. In addition, from a methodological standpoint, efforts to improve reproducibility increasingly emphasize standardization of sulcal labeling and the development of automated solutions aimed at limiting rater-dependent variability. Notably, recent self-supervised learning frameworks have shown encouraging potential for identifying ACC folding patterns, pointing to a more objective sulcal-pattern characterization (Dufournet et al. 2025).

Generally, from a purely qualitative point of view, as already noted (Santacroce et al. 2024), the right hemisphere seems to have a clearer morphology, making it easier to detect morphological patterns with respect to its left counterpart. This may partly account for the fewer discrepancies observed among the comparison studies in the right hemisphere. It would be interesting to investigate the possible causes of this inter-hemispheric difference, possibly due to genetic factors related to cortical development. The phenomenon known as the Yakovlevian torque, i.e., the greater frontal extension of the right hemisphere and occipital extension of the left hemisphere along the medial line (LeMay et al. 1976), could be involved. In particular, the greater superficial extension of the right compared to the left frontal lobe may allow for less convoluted cortical folding and clearer sulcus morphological patterns. Furthermore, the notion that the right hemisphere develops earlier than the left and is therefore less influenced by the in-utero environment during fetal life (Geschwind and Lateralization 1985, Sun et al. 2005) supports the idea that its sulcal morphology stabilizes at an early stage, which may explain why it appears easier to define. However, further research is needed to clarify this aspect.

While this study provides valuable insights for understanding the impact of ACC structural morphology on DD, it also raises questions about the broader neural mechanisms underlying DD. Future research should investigate whether ACC asymmetry interacts with other structural and functional features, such as white matter integrity or neural connectivity, to influence decision-making. It would also be interesting to investigate whether the sulcal pattern of other regions involved in DD additionally contributes to the effect of the ACC sulcal morphology. Moreover, longitudinal studies could clarify whether ACC asymmetry drives the development of delay discounting behavior, particularly in the case of disease onset, and shed light on the causal pathways involved.

In summary, the present study highlights the critical role of cortical morphology, in particular the anterior cingulate cortex (ACC) sulcal asymmetry, in shaping individual differences in DD behavior. By demonstrating this sulcal morphological effect and its dissociation from unrelated cognitive domains or cortical regions, our findings suggest that stable anatomical features may serve as robust markers of impulsivity. The structural asymmetry of the ACC, likely reflecting interhemispheric specialization, not only advances our understanding of decision-making but also opens new avenues for personalized approaches to cognitive enhancement and clinical intervention. Sulcal patterns can be regarded as a ‘fossil trace’ of early brain development, influencing later cognitive and functional adaptations. As such, they may act as potential neuroprotective or risk factors. This perspective enriches our interpretation of sulcal morphology as not just a static anatomical feature but as a persistent signature of developmental and functional optimization. These ‘fossilized’ features offer a unique window into the neural substrates of behavior and provide a foundation for future research aimed at disentangling the complex interplay between brain structure, function, and cognition.

## Supplementary Information

Below is the link to the electronic supplementary material.

Supplementary Material 1

## Data Availability

No datasets were generated or analysed during the current study.
