# Supplementary material for: Asymmetry of the sulcal pattern of the anterior cingulate cortex modulates delay discounting
Source: Brain Struct Funct. 2026 Apr 27;231(4):55. doi: 10.1007/s00429-026-03114-8 (PMC13111510; doi:10.1007/s00429-026-03114-8)
Supplement: Supplementary file 1 — Supplementary Material 1 [file 429_2026_3114_MOESM1_ESM.docx]

Supplementary Analyses

**Characterization of the CS sulcal pattern and relation with DD**

For each hemisphere, the CS was classified as continuous when it formed a single uninterrupted sulcus and as interrupted when it was split into two or more segments. The CS interruption was classified as symmetric when both hemispheres showed the same pattern (both continuous or both interrupted) and as asymmetric when the pattern differed between hemispheres (continuous in one hemisphere and interrupted in the other). The inter-rater reliability yielded an agreement of 87.44% regarding the presence of the continuous vs. interrupted ACC pattern for both the left and right hemispheres. After that, all discrepant or doubtful cases (N=49, for each hemisphere) were re-examined by a third expert (GC).

The ‘continuous’ type was observed in 61.03% of right hemispheres and 65.90% of left hemispheres, whereas the ‘interrupted’ type was found in 38.97% of right hemispheres and 34.10% of left hemispheres. In addition, the ‘symmetry’ and ‘asymmetry’ patterns were observed in 47.95% and 52.05% of cases, respectively.

To investigate whether a distinct morphological feature of the Cingulate Cortex (i.e., the interruption of CS) was associated with DD rate, we performed a Generalized Linear Model (GLM) including the CS pattern (continuous/interrupted) and sex as categorical factors and the third factor of the PCA (delay discounting) as the dependent variable. The analysis revealed no significant association in either the right (β = 0.0100, SE = 0.052, z = -0.192, p = 0.847) or left (β = -0.0700, SE = 0.056, z = -1.2422, p = 0.214) hemisphere.

In addition, we classified the symmetry/asymmetry pattern of the cingulate sulcus based on interruption and included symmetry/asymmetry of the CS pattern and sex as categorical factors, and the DD factor as dependent variable. No significant result was found (β = 0.0171, SE = 0.101, z = 0.168, p = 0.866).

**Supplementary Table 1. Post hoc chi-square analysis of frequency distribution for ACC patter (single vs parallel).**

In this table, Study 1 corresponds to Yucel et al. (2001), Study 2 to Cachia et al. (2014), Study 3 to Borst et al. (2014), Study 4 to Tissier et al. (2018), and Study 5 represents the Present study.

| **Contrast** | **p-value** | **corrected p-value** |
| --- | --- | --- |
| **LEFT** | | |
| Study 1 vs 2 | 0,0213 | 0,2128 |
| Study 1 vs 3 | 0,9479 | 1 |
| Study 1 vs 4 | <0,001 | <0,001 |
| Study 1 vs 5 | 0,08446 | 1 |
| Study 2 vs 3 | 0,0122 | 0,1219 |
| Study 2 vs 4 | <0,001 | <0,001 |
| Study 2 vs 5 | 0,0489 | 0,4893 |
| Study 3 vs 4 | <0,001 | 0,0001 |
| Study 3 vs 5 | 0,6871 | 1 |
| Study 4 vs 5 | <0,001 | <0,001 |
| **RIGHT** | | |
| Study 1 vs 2 | 0,6982 | 1 |
| Study 1 vs 3 | 0,0237 | 0,2374 |
| Study 1 vs 4 | 0,0534 | 0,5344 |
| Study 1 vs 5 | 0,1630 | 1 |
| Study 2 vs 3 | 0,0839 | 0,8391 |
| Study 2 vs 4 | 0,1626 | 1 |
| Study 2 vs 5 | 0,0532 | 0,5323 |
| Study 3 vs 4 | 0,8484 | 1 |
| Study 3 vs 5 | 0,0002 | 0,0017 |
| Study 4 vs 5 | 0,0006 | 0,0058 |
| **SYMMETRY/ASYMMETRY** | | |
| Study 1 vs 2 | 0,9142 | 1 |
| Study 1 vs 3 | 0,5686 | 1 |
| Study 1 vs 4 | <0,001 | 0,0001 |
| Study 1 vs 5 | 0,6094 | 1 |
| Study 2 vs 3 | 0,7495 | 1 |
| Study 2 vs 4 | <0,001 | <0,001 |
| Study 2 vs 5 | 0,4467 | 1 |
| Study 3 vs 4 | <0,001 | <0,001 |
| Study 3 vs 5 | 0,2216 | 1 |
| Study 4 vs 5 | 0,0001 | 0,0009 |

**Supplementary Table 2. Association between ACC morphology and single PCA’s tasks/metrics included in the HCP.** The table reports the β values, SE, z points, and uncorrected p values of ACC symmetry/asymmetry pattern and all tasks entered into the PCA. ACC= accuracy, RT= reaction time; AUC= area under the curve; C= specificity.

| **Measure** | **Metric** | **β** | **SE** | **z** | **p** |
| --- | --- | --- | --- | --- | --- |
| **Non-Verbal Episodic Memory** |  |  |  |  |  |
| Picture Sequence Memory | ACC | 0.5237 | 1.337 | 0.392 | 0.695 |
| **Verbal Episodic Memory** |  |  |  |  |  |
| Penn Word Memory Test (Form A) | ACC | 0.2574 | 0.293 | 0.878 | 0.380 |
| Penn Word Memory Test (Form A) | RT | 31.8251 | 29.531 | 1.078 | 0.281 |
| **Working Memory** |  |  |  |  |  |
| List Sorting Working Memory Test | ACC | -2.1017 | 1.096 | -1.918 | 0.055 |
| **Executive Function** |  |  |  |  |  |
| Dimensional Change Card Sort Test | ACC | 0.8346 | 0.048 | 0.797 | 0.429 |
| Flanker Inhibitory Control and Attention Test | ACC | 0.3552 | 1.006 | 0.353 | 0.724 |
| **Fluid Intelligence** |  |  |  |  |  |
| Penn Progressive Matrices | ACC | -0.0098 | 0.465 | -0.021 | 0.983 |
| Penn Progressive Matrices | RT | 1527.8966 | 902.039 | 1.694 | 0.090 |
| **Language** |  |  |  |  |  |
| Picture Vocabulary Test | ACC | -0.9938 | 1.083 | -0.917 | 0.359 |
| Oral Reading Recognition Test | ACC | 0.6344 | 0.980 | 0.648 | 0.517 |
| **Processing Speed** |  |  |  |  |  |
| Pattern Comparison Processing Speed Test | ACC | **-4.2859** | **1.523** | **-2.815** | **0.005** |
| **Self-Regulation/Impulsivity** |  |  |  |  |  |
| DelayDiscounting_200 | AUC | **0.0429** | **0.021** | **2.033** | **0.042** |
| DelayDiscounting_40 | AUC | 0.0357 | 0.028 | 1.269 | 0.205 |
| **Spatial Orientation** |  |  |  |  |  |
| Variable Short Penn Line Orientation Test | ACC | 0.8357 | 0.441 | 1.897 | 0.058 |
| Variable Short Penn Line Orientation Test | RT | 25.5921 | 37.834 | 0.676 | 0.499 |
| **Sustained Attention** |  |  |  |  |  |
| Short Penn Continuous Performance | RT | 5.9568 | 4.143 | 1.438 | 0.151 |
| Short Penn Continuous Performance | d’ | -0.0012 | 0.005 | -0.242 | 0.809 |
| Short Penn Continuous Performance | C | 0.0010 | 0.004 | 0.260 | 0.794 |

Task descriptions are based on the HCP S1200 Reference Manual (Van Essen et al., 2013; Barch et al., 2013; HCP S1200 Release Manual).

**Non‑Verbal Episodic Memory.**

- Picture Sequence Memory Test (NIH Toolbox). This task assesses non‑verbal episodic memory by requiring participants to recall and reconstruct the order of visually presented picture sequences.

**Verbal Episodic Memory.**

- Penn Word Memory Test (Penn CNB). This recognition memory task requires participants to identify previously presented words among novel distractors.

**Working Memory.**

- List Sorting Working Memory Test (NIH Toolbox). This task evaluates working memory by asking participants to maintain and manipulate information while ordering items according to specified criteria.

**Executive Function.**

- Dimensional Change Card Sort Test (NIH Toolbox). This measure assesses cognitive flexibility by requiring participants to switch between sorting rules.
- Flanker Inhibitory Control and Attention Test (NIH Toolbox). This task evaluates inhibitory control and selective attention by requiring responses to target stimuli in the presence of congruent or incongruent flankers.

**Fluid Intelligence.**

- Penn Progressive Matrices (Penn CNB). This measure assesses abstract reasoning through the selection of the missing element in visual matrices of increasing difficulty.

**Language.**

- Picture Vocabulary Test (NIH Toolbox). This task measures receptive vocabulary and language comprehension.
- Oral Reading Recognition Test (NIH Toolbox). This measure assesses reading decoding ability through accurate pronunciation of printed words.

**Processing Speed.**

- Pattern Comparison Processing Speed Test (NIH Toolbox). This task evaluates visual processing speed by requiring rapid comparison of simple visual patterns.

**Self‑Regulation / Impulsivity.**

- Delay Discounting Task (Penn CNB). This measure assesses impulsive decision‑making by quantifying preferences for smaller immediate rewards versus larger delayed rewards.

**Spatial Orientation.**

- Variable Short Penn Line Orientation Test (Penn CNB). This task evaluates spatial orientation by requiring participants to match the orientation of line pairs.

**Sustained Attention.**

- Short Penn Continuous Performance Test (Penn CNB). This measure assesses sustained attention through the detection of target stimuli presented in a continuous sequence.
